# Supplementary material for: Canonical and phosphoribosyl ubiquitination coordinate to stabilize a proteinaceous structure surrounding the Legionella-containing vacuole
Source: bioRxiv. 2025 Jul 23:2025.07.22.666189. Preprint. [Version 1] doi: 10.1101/2025.07.22.666189 (PMC12330589; doi:10.1101/2025.07.22.666189)
Supplement: 1 [file NIHPP2025.07.22.666189v1-supplement-1.pdf]

bioRxiv preprint doi: https://doi.org/10.1101/2025.07.22.666189; this version posted July 23, 2025. The copyright holder for this preprint (which was not certified by peer review) is the author/funder, who has granted bioRxiv a license to display the preprint in perpetuity. It is made available under aCC-BY 4.0 International license.

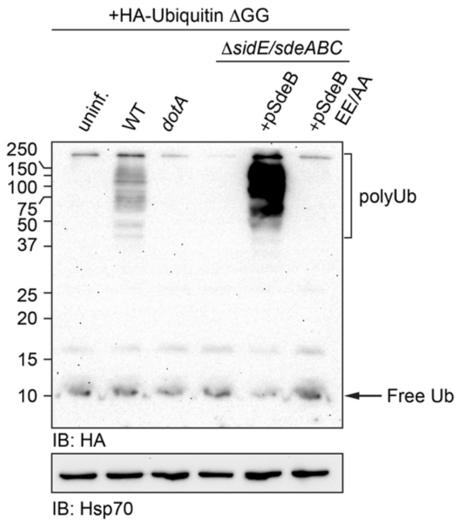

Figure S1 (related to Figure 2): SdeB EE/AA is unable to catalyze PR-ubiquitination.

Western blot analysis of HA-Ub ΔGG conjugation in whole cell lysates prepared from cells infected with the indicated *L.p.* strain for 1 hour.

bioRxiv preprint doi: https://doi.org/10.1101/2025.07.22.666189; this version posted July 23, 2025. The copyright holder for this preprint (which was not certified by peer review) is the author/funder, who has granted bioRxiv a license to display the preprint in perpetuity. It is made available under aCC-BY 4.0 International license.

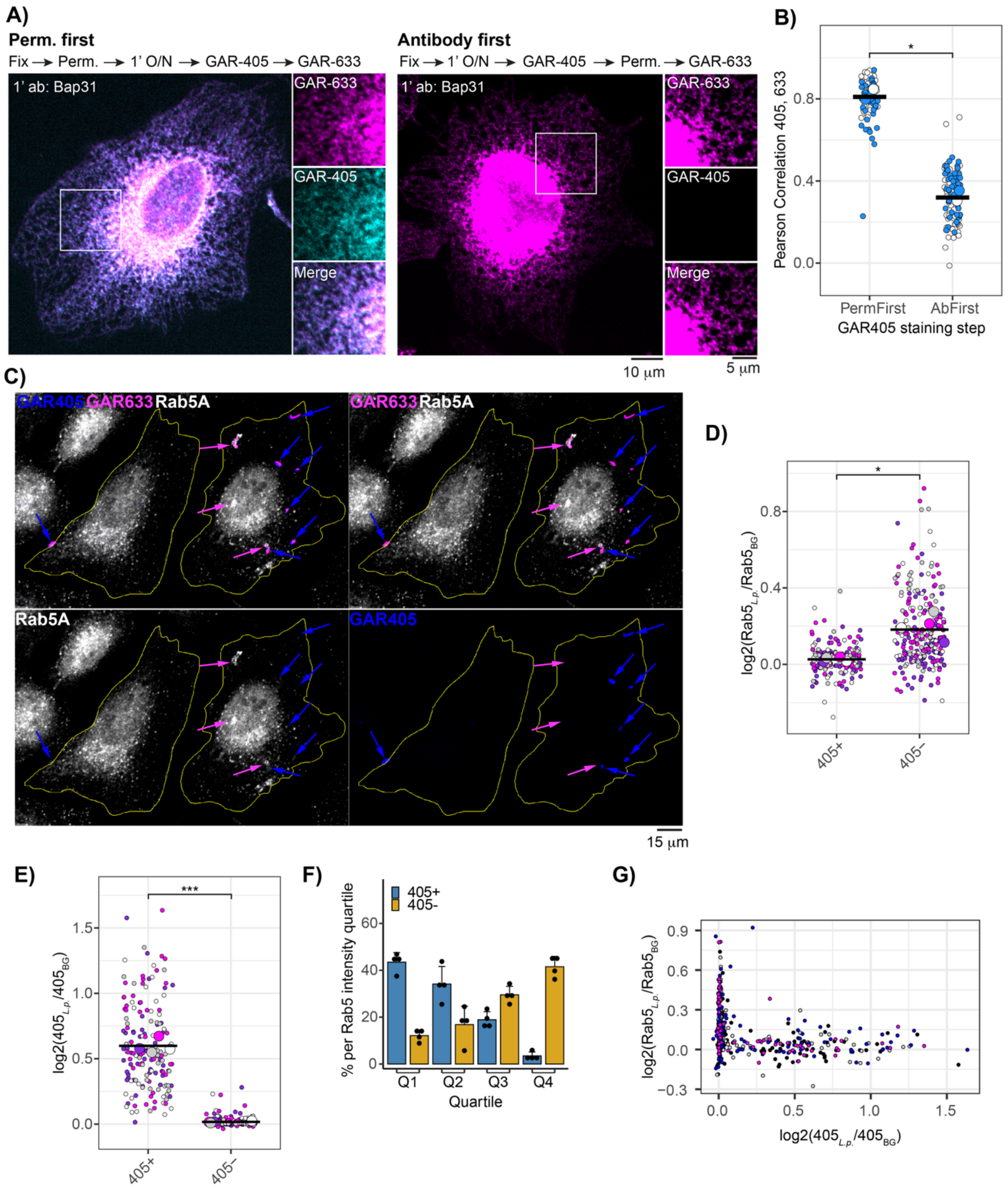

Figure S2: Validation of dual-stain method for detection of extracellular bacteria.

(A) Representative images of cells permeabilized either before or after Bap31 primary antibody and GAR-405 staining. (B) Pearson correlation of GAR-633 and GAR-405 signal in cells prepared as in flowchart shown in A. N=2, 50 cells analyzed per replicate per condition. Welch's two-sample t-test, p = 0.006. (C) Representative images of cells infected with *L.p.* WT and processed using

bioRxiv preprint doi: https://doi.org/10.1101/2025.07.22.666189; this version posted July 23, 2025. The copyright holder for this preprint (which was not certified by peer review) is the author/funder, who has granted bioRxiv a license to display the preprint in perpetuity. It is made available under aCC-BY 4.0 International license.

dual stain method for the *L.p.* opsonization antibody, as well as immunofluorescence analysis of endogenous Rab5. Double stained (extracellular) bacteria are indicated by blue arrows, whereas single stained (intracellular) bacteria are indicated by magenta arrows. (D) Quantification of normalized Rab5 intensity at either double (405+) or single (405-) stained bacteria. N = 4, ~30-90 bacteria scored per replicate per condition. Welch's two sample t-test, p = 0.0127. (E) Measurement of normalized GAR405 intensity at bacterial cell bodies for same dataset described in D. Welch's t-test, p = 0.0002. (F) Distribution of Rab5 intensity values for dataset described in D. For each replicate, data was pooled, and the percentage of 405+ versus 405- bacteria falling into each quartile was tabulated. (G) Normalized GAR405 intensity vs. normalized Rab5 intensity for dataset described in D. Note that very few datapoints show strong signal for both Rab5 and GAR405. Color represents biological replicate (N=4), 418 total bacteria scored.

bioRxiv preprint doi: https://doi.org/10.1101/2025.07.22.666189; this version posted July 23, 2025. The copyright holder for this preprint (which was not certified by peer review) is the author/funder, who has granted bioRxiv a license to display the preprint in perpetuity. It is made available under aCC-BY 4.0 International license.

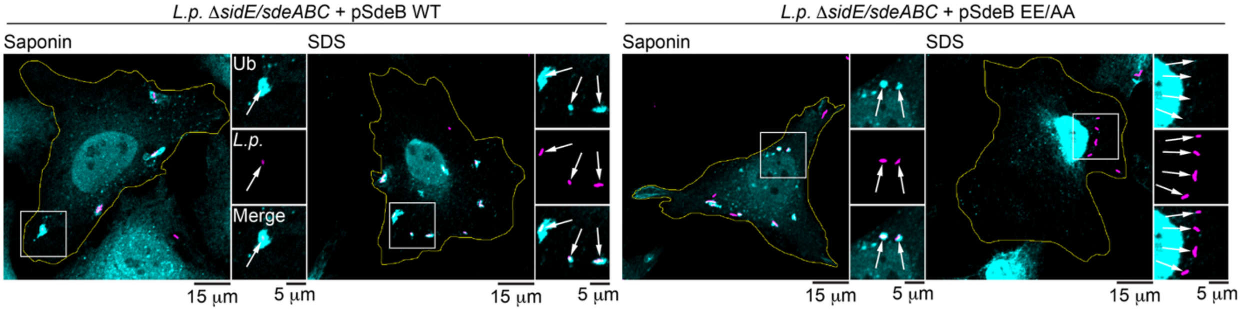

Figure S3 (related to Figure 5): Extended representative images of ubiquitin detergent resistance.

Endogenous ubiquitin staining in cells infected with the indicated strain and permeabilized with either saponin or SDS at 1hpi.

bioRxiv preprint doi: https://doi.org/10.1101/2025.07.22.666189; this version posted July 23, 2025. The copyright holder for this preprint (which was not certified by peer review) is the author/funder, who has granted bioRxiv a license to display the preprint in perpetuity. It is made available under aCC-BY 4.0 International license.

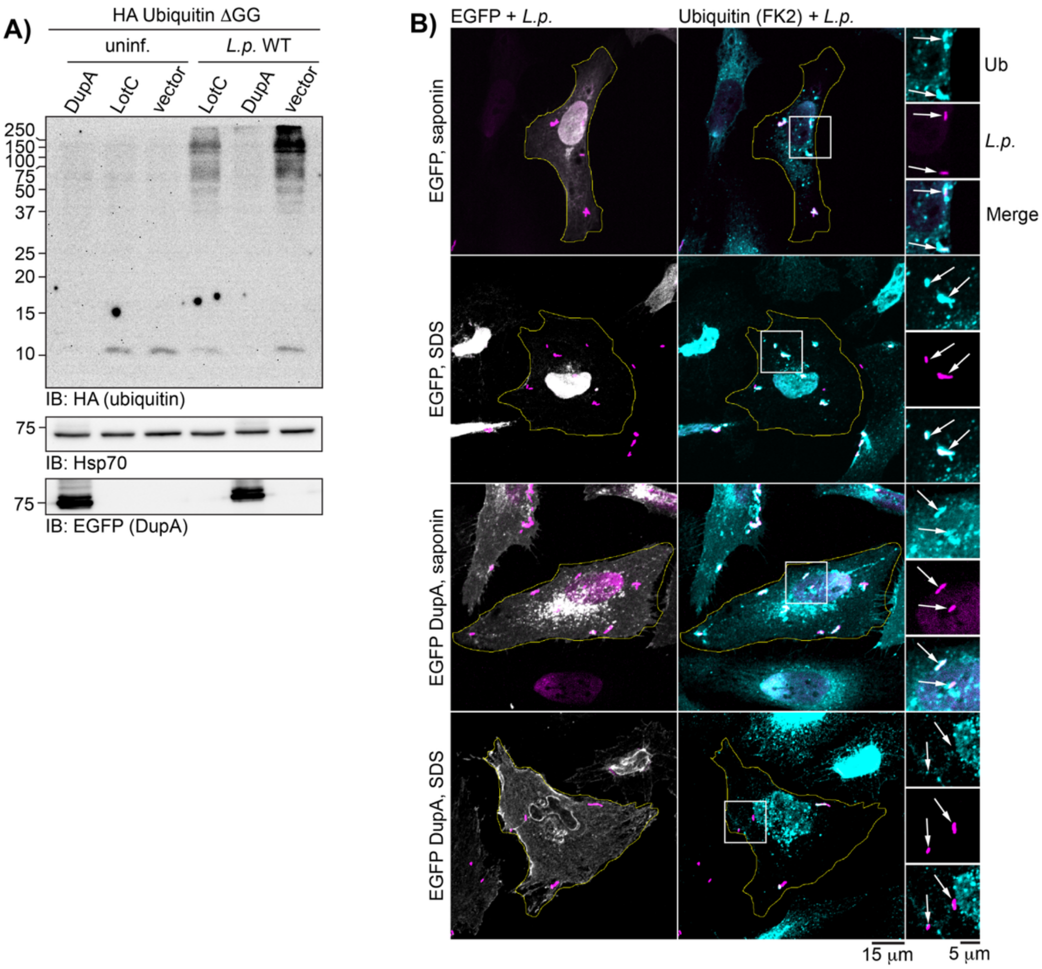

Figure S4 (related to Figure 6): Ectopically expressed EGFP-DupA is active against PR-ubiquitin conjugates.
(A) Western blot analysis of lysates from cells transfected with HA-ubiquitin ΔGG and the indicated construct (LotC is a canonical ubiquitin ligase effector), and either left uninfected or infected with *L.p.* WT. (B) Representative images of endogenous ubiquitin staining in cells transfected with either EGFP alone or EGFP-DupA, infected with *L.p.* WT, and permeabilized with either sapoinin or SDS. For all infection experiments, cells were fixed or lysed at 1hpi.

bioRxiv preprint doi: https://doi.org/10.1101/2025.07.22.666189; this version posted July 23, 2025. The copyright holder for this preprint (which was not certified by peer review) is the author/funder, who has granted bioRxiv a license to display the preprint in perpetuity. It is made available under aCC-BY 4.0 International license.

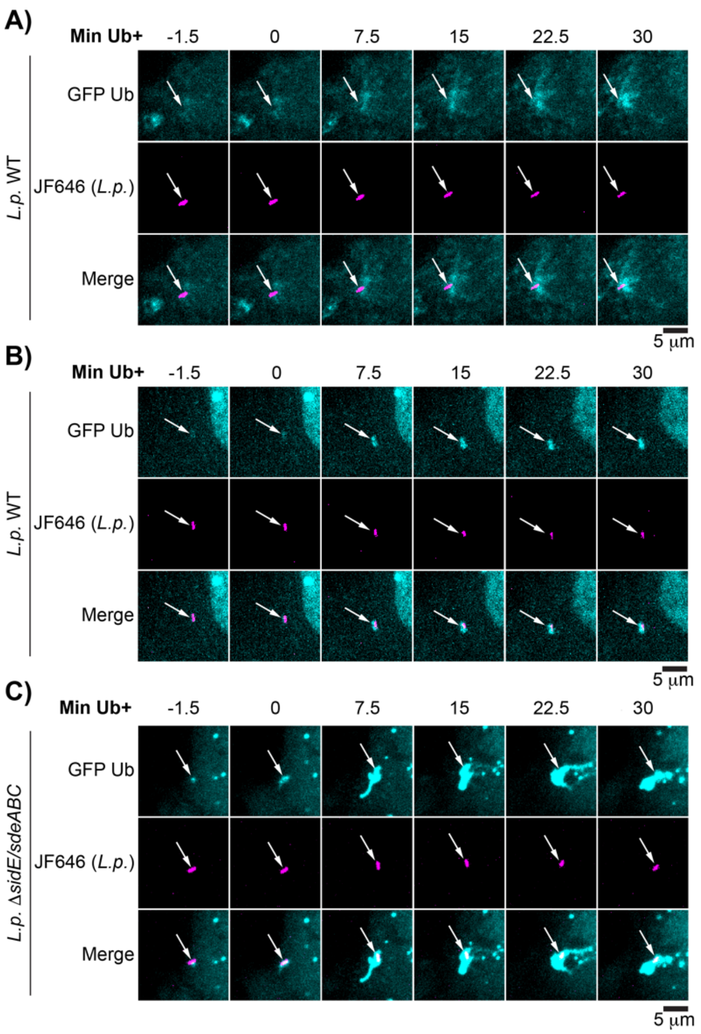

Figure S5 (related to Figure 7): Uncommon LCV-associated ubiquitin morphology patterns observed during live imaging.

Example cases of (A) diffuse ubiquitin localization throughout imaging at WT LCV, (B) compact ubiquitin localization at WT LCV, and (C) highly dynamic compact-to-expansive ubiquitin morphology at the SidE family knockout strain LCV.

Supplemental movies S1-4

See Methods for sample preparation and image acquisition. Scale bar length corresponds to 10 µm.
